# Progressive Applications of Hyperbranched Polymer Based on Diarylamine: Antimicrobial, Anti-Biofilm and Anti-Aerobic Corrosion

**DOI:** 10.3390/ma13092076

**Published:** 2020-04-30

**Authors:** Khalid I. Kabel, Ahmed Labena, Mohamed Keshawy, Wael N. Hozzein

**Affiliations:** 1 Petroleum Applications Department, Egyptian Petroleum Research Institute (EPRI), Nasr City 11727, Cairo, Egypt; drkhalid1977@epri.sci.eg (K.I.K.); elkeshawy2006@epri.sci.eg (M.K.); 2 Processes Development Department, Egyptian Petroleum Research Institute (EPRI), Nasr City 11727, Cairo, Egypt; 3 Bioproducts Research Chair, Zoology Department, College of Science, King Saud University, Riyadh 11451, Saudi Arabia; whozzein@ksu.edu.sa; 4 Botany and Microbiology Department, Faculty of Science, Beni-Suef University, Beni-Suef 62511, Egypt

**Keywords:** hyperbranched polymer, AB_2_ monomer approach, antimicrobial activity, antibiofilm, corrosion inhibitor

## Abstract

New generations of hyperbranched aramids were synthesized from diarylamine and methyl acrylate using an AB_2_ monomer approach in a straightforward one-pot preparation. The chemical structure of hyperbranched Phenylenediamine/Methyl Acrylate HB(PDMA was confirmed by Fourier Transform Infrared (FTIR) and Nuclear Magnetic Resonance (^1^HNMR) spectroscopy. In addition, the particle’s size and distribution were recorded using Dynamic Light Scattering (DLS). Moreover, the synthesized HB(PDMA)s displayed broad-spectrum antimicrobial activities against Gram-positive and Gram-negative bacteria as well as yeast strains and anti-biofilm activity where the highest activity was attributed to HB(PDMA)G_4_ at the lowest Minimum Inhibitory, Minimum Bactericidal, and Fungicidal Concentrations (MIC, MBC, and MFC, respectively). Furthermore, the HB(PDMA)s expressed anti-bacterial activity against isolated *Pseudomonas* sp. (R301) at a salinity of 35,000 ppm (NaCl). In addition, they revealed different corrosion inhibition efficiencies at the cultivated medium salinity at the estimated minimum bactericidal concentrations. The highest metal corrosion inhibition efficiencies were 59.5 and 94.3% for HB(PDMA)G_4_ at the Minimum Bactericidal Concentrations (MBCs) and two times Minimum Bactericidal Concentrations (2XMBCs), respectively, in comparison to both negative and positive controls.

## 1. Introduction

According to their unique chemical and physical properties, hyperbranched polymers (HBP)s have recently been of special interest [1], which can be attributed to their one-pot synthetic route on a large scale when compared to the multiple step synthetic-route of dendrimers. HBPs have low viscosity, good solubility, plentiful of functional groups, and a globular shape [2,3]. Furthermore, hyperbranched polymers have various applications in many fields [4] including chemistry, life science, biotechnology, and the petroleum industry. Their applications in the petroleum industry include demulsifiers [5], corrosion and scale inhibitors [6], oil spill dispersants [7], and asphaltene dispersants [8]. HBPs consist of a central core spread out to form a network of repeating branches ended by characteristic groups, which form an external surface of the polymeric nanoparticle. Their properties depend on the structural characteristics of the core, the branches, and the end-reactive groups [9]. Moreover, HBPs have promising merits as antimicrobial agents. It is highly remarkable that polymeric compounds with specific functional groups as thiols, amines, phenols, and esters have high potential to resist either fungal or bacterial Gram-positive and Gram-negative bacterial strains [10,11]. The polymerization routes, polycondensation, ring opining, or polyaddition have been used to synthesize HBPs such as aromatic polyesters, polyurethanes, and aromatic polyamides [12,13]. The polycondensation route of AB_x_ monomers has been early proposed by Flory [14]. Furthermore, Gunatillake et al. reported the first preparation of hyperbranched polymer using such routes [15]. Afterward, the synthesis of hyperbranched aramid copolymers by direct polymerization and their properties as well as the effect of the monomer ratio on the mechanical properties were studied by Jikei et al. [16]. Kakimoto et al. [17] prepared hyperbranched aromatic polyamides of the AB_2_ monomer using direct polymerization and thermal methods. Recently, a commercial diamine and triacid were used to synthesize hyperbranched aramids, in addition to many new synthetic approaches that have been previously reported [18]. However, *p*-phenylenediamine is considered as one of the most investigated conducting polymers and has attracted much interest in many studies with various practical applications with relation to its high conductivity, outstanding air stability, and special physical–chemical properties compared with other conducting polymers [19]. Many phenylenediamines have been applied as corrosion inhibitors of mild steel [20,21]. It is well known that biocorrosion or Microbially-Influenced Corrosion (MIC) is affected by microbial activities, especially when the organisms are in contact with the metal surface to form a biofilm. Indeed, microbial cells are the building blocks of the biofilms and ease the attachment of cells with a metal surface, changing the electrochemical processes at their interface, thus referring to the extracellular polymeric substances (EPS) [22]. The objective of the present work was to synthesize hyperbranched polymer-aramids using the AB_2_ monomer approach, *p*-phenylenediamine, and methyl acrylate in three generations (G_2_, G_3_, G_4_) as an easy one-pot preparation. Moreover, the efficiency of the synthesized HB(PDMA)s were investigated as antimicrobial agents against Gram-positive, Gram-negative bacteria, and yeast strains, and as anti-biofilms (bacterial adhesion) agents. The HB(PDMA)s were evaluated as biocides and corrosion inhibitors against *Pseudomonas* sp. (R301) at a salinity of 35,000 ppm (NaCl).

## 2. Materials and Methods

### 2.1. Materials

*p*-phenylenediamine (PD), methyl acrylate (MA), dimethylformamide (DMF), p-toluensulfonic acid (PTSA), tetrahydrofuran (THF) Gel Permeation Chromatography (GPC) grade, and methanol were obtained from Sigma Aldrich (St. Louis, MO, USA) and used without further purification. All other chemicals were reagent grade unless otherwise described.

### 2.2. Synthesis of Hyperbranched Phenylenediamine/Methyl Acrylate HB(PDMA)

**Figure 1:**
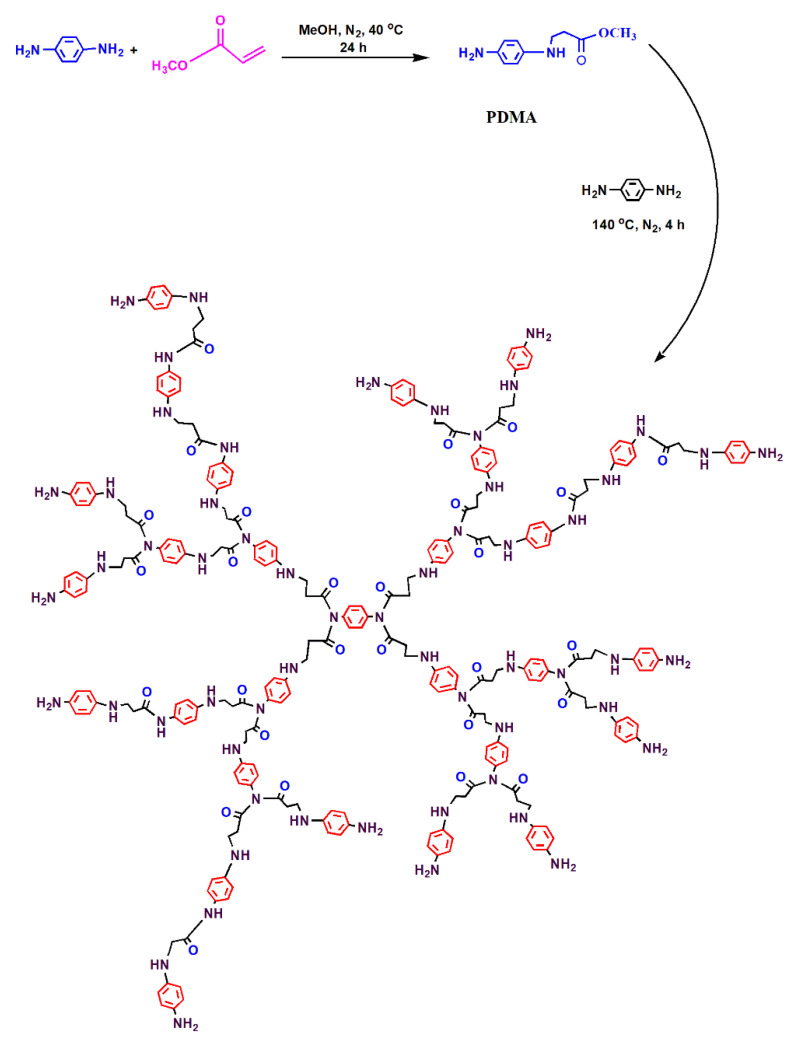
Reaction scheme of the synthesized HB(PDMA)s.

In two necked flasks, 10.8 g (0.1 mol) of *p-*phenylenediamine (PD) was dissolved in 50 mL methanol, and a solution of 12 g (0.125 mol) of methyl acrylate (MA), dissolved in 20 mL of methanol, was added dropwise to the PD solution with continuous stirring under a nitrogen atmosphere for 4 h at 0 °C, and for 24 h at room temperature. The product was purified and evaporated in a vacuum oven at 40 °C to obtain the PDMA monomer. Then, 1.08 g (0.01 mol) of PD (as a core) was dissolved in 40 mL of DMF solvent at room temperature and placed in three necked flasks fitted with a thermometer, dean-stark with condenser, and dropping funnel. Then, 11.64 g (0.16 mol) of the prepared PDMA monomer was added with continuous stirring under a nitrogen atmosphere at 140 °C for 4 h in the presence of 0.1% PTSA (as a catalyst). The product was purified, and the solvent was evaporated in a rotary to obtain HB(PDMA)G_2_. The reaction was repeated using 23.28 g (0.32 mol) and 46.56 g (0.64 mol) of the PDMA monomer to obtain HB(PDMA)s G_3_ and G_4_, respectively. The reaction scheme is illustrated in Figure 1.

### 2.3. Characterization of the Prepared Hyperbranched Polymers

The chemical structures were recorded using a Nicolet iS10 FTIR spectrophotometer (Thermo Fisher Scientific, Waltham, MA, USA) in a wavenumber range of 500–4000 cm^−1^. The ^1^HNMR spectroscopy was also recorded using a JEOL ECA-500 II spectrometer (500 MHz, JEOL Ltd. Co., Tokyo, Japan) with deuterated dimethyl sulfoxide (DMSO-d_6_) as a solvent. Dynamic light scattering (DLS) was conducted to measure the particle size distribution by using a Zetasizer Nano ZS90 (Malvern Co., Malvern, UK) with a laser angle of 90° at 25 °C. Gel permeation chromatography (GPC) (Waters 515-2410, Waters, USA) was conducted on a GPC-Water 2410 at 40 °C with a refractive index detector using four columns of Styragel HR THF 7.8 × 300 mm, as calibrated by the Polystyrene Ready Cal Standards. The mobile phase was THF with a flow rate of 1 mL/min.

### 2.4. Antimicrobial Activity Test of the Synthesized HB(PDMA)s

#### 2.4.1. Microbial Strain Standards

*Staphylococcus aureus* (ATCC^®^ 29737™), *Bacillus subtilis* (ATCC^®^ 6633™), *Escherichia coli* (ATCC^®^ 8739™), and *Candida albicans* (ATCC^®^ CRM10231™) were collected from the American Type Culture Collection (Rockville, MD, USA).

#### 2.4.2. Microbial Media and Cultivation Conditions

The bacterial strains were cultivated and enumerated on Mueller Hinton Broth (MHB) or Mueller Hinton Agar (MHA) (Difco, Franklin Lakes, NJ, USA) at 37 °C with an incubation period of 24 h. Furthermore, Sabouraud Dextrose Broth (SDB) or Sabouraud Dextrose Agar (SDA) (Difco, Sparks, MD, USA) were used for the cultivation and enumeration of the *Candida albicans* strain at 30 °C for a period of incubation of 48 h.

The antimicrobial activity of the synthesized HB(PDMA)s G_2_, G_3_, G_4_ at a concentration of 10 millimoles (mM) were estimated using a method of agar well (10 mm) diffusion (as previously reported [23]) on MHA and SDA plates for the bacterial and the yeast strains, respectively. After overnight cultivation at 37 °C for the bacterial strains and 48 h at 30 °C for the *Candida albicans* strain, the biological activity was then evaluated by measuring the clearing zone’s diameter. All tests were performed three times, and the average values were recorded. Furthermore, sterile water was used as a negative control and standard antimicrobial agents’ Amoxicillin (100 ppm), Tetracycline (100 ppm), and Fluconazole (100 ppm) were used as positive controls.

#### 2.4.3. Minimum Inhibitory Concentrations (MIC)s and Minimum Bactericidal/Fungicidal Concentrations (MBC/MFC)s of the Synthesized HB(PDMA)s

The Minimum Inhibitory Concentration (MIC) “is defined as the lowest concentration of an antimicrobial agent that inhibits the development of visible microbial growth” [24]. However, the Minimum Bactericidal/Fungicidal Concentration (MBC/MFC) “is the lowest concentration of an antimicrobial agent required for killing 99% of the germ” [25]. The MICs were determined by the two-fold method in 96-well microtiter plates [26,27]. Briefly, the bacteria and yeast strains-inocula were prepared according to the Clinical Laboratory Standards Institute (CLSI) [28,29]. The bacterial and yeast strains were refreshed twice on MHB at 37 °C for an 18 h incubation period and on SDB at 30 °C for a 48 h incubation period, respectively, after 50% glycerol media reservation. The cultivated bacteria and yeast cells were checked for their purity using MHA and SDA, respectively. Then, the experimental bacterial inocula proceeded. After a purity check, 3–5 separate colonies were collected from the MHA plates and re-cultivated on 10 mL MHB at 37 °C for an overnight incubation period with continuous agitation at 200 rpm. Afterward, the overnight cultures were used as inocula for 10 mL MHB by adjusting their optical density (OD) to 0.2 at 550 mm. The 10 mL MHB cultures were further incubated at 37 °C for 3–4 h (i.e., until they achieved an OD550 of 1–2 under agitation of 200 rpm). Next, the MHB cultures were then diluted using fresh media until obtain an OD550 of 0.2. The experimental bacterial-inocula were readied by diluting the obtained cultivated cultures by 100 dilution folds (DF) and 1000 dilution folds for Gram-positive and Gram-negative bacteria, respectively, to achieve bacterial counts of 1–2 × 10^8^ CFU/mL (colony-forming unit/mL) for Gram-positive bacteria and 1–2 × 10^9^ CFU/mL for Gram-negative bacteria, according to the CLSI [28]. The experiment yeast inoculum was then prepared. After a purity check, 3–5 cells were collected from the SDA plate and allowed to grow on 10 mL SDB at 30 °C for 24 h under continuous agitation at 200 rpm. Afterward, the cultivated cells were diluted by adjusting the OD to 550 to 0.2 according to the CLSI [29], which corresponded to the *Candida albicans* count of 5 × 10^6^ CFU/mL. We serially diluted 100 µL of the synthesized HB(PDMA)s G_2_, G_3_, and G_4_ (at a concentration of 10 mM) using MHB for the bacterial strains and SDB for the yeast strain onto the micro-titer plates (Nunc GmbH & Co., Wiesbaden, Germany) and further inoculated them with 100 µL of the microbial suspension. There were two controls: positive (microbially inoculated without the synthesized HB(PDMA)s) and negative (only sterile media). After that, the micro-titer plates were incubated under aerobic conditions for an incubation period of 20 h at 37 °C for the bacterial strains and a 48 h incubation period for the *Candida albicans* strain at 30 °C. In order to visualize the results, 30 µL of 0.01% resazurin (HiMedia) solution was added to each well [30], and the plates were further incubated. The changing of the well’s color to pink indicates a reduction of resazurin (indicates microbial growth). MBC/MFC values of the synthesized HB(PDMA)s G_2_, G_3_, and G_4_ were estimated by taking 10 µL from the wells (before adding the resazurin indicator) that showed no visible growth and were further sub-cultured onto agar media (MHA, SDA plates) [31]. The agar plates were then incubated at 37 °C for 20 h for the bacterial species and at 30 °C for 48 h in the case of *C. albicans* (i.e., until growth was seen in the control plates). MBC/MFC values were defined as the corresponding concentrations of the synthesized HB(PDMA)s needed to indicate 99.5% killing.

### 2.5. Anti-Biofilms (Bacterial Adhesion) Activity of the Synthesized HB(PDMA)s and Minimum Biofilm Inhibitory Concentrations (MBICs) Detection

A semi-quantitative adherence assay on 96-well tissue culture plates was used to study the anti- biofilms (the bacterial adhesion of *B. subtilis* and *E. coli*) of the synthesized HB(PDMA)s G_2_, G_3_, and G_4_, as previously reported [32]. The bacterial inocula (100 µL) were prepared according to the CLSI method [30] and started with approximate concentrations of 1–2 × 10^8^ CFU/mL and 1–2 × 10^9^ CFU/mL for *B. subtilis* and *E. coli*, respectively. We serially diluted 100 µL of the synthesized HB(PDMA)s G_2_, G_3_, and G_4_ (supplemented with 1% Glucose) onto the micro-titer plates. The test was performed parallel with positive (inoculated well without the synthesized HB(PDMA)s) and negative (only sterile media) controls. The plates were then incubated for 20 h at 37 °C. The plates were further cleaned three times with 200 μL of 1× phosphate buffer saline (PBS) at pH 7.4, dried, fixed with ethanol, then subsequently stained with 0.1% Crystal Violet (Merck, Darmstadt, Germany). Afterward, the plates were washed again and further dried for 2 h [27]. The developed positive result appeared as purple rings that formed on the well’s bottom and side. The minimum biofilm inhibitory Concentrations (MBICs) were calculated as the lowest concentration of the synthesized HB(PDMA)s G_2_, G_3_, and G_4_ that inhibited the development of visible microbial growth adherence (biofilm) on MHB (supplemented with 1% Glucose) after an incubation period.

### 2.6. Antimicrobial Activity Test of the Synthesized HB(PDMA)s against Isolated Slime Forming Bacteria (SFB) Cultivated at High Salinity (35,000 ppm NaCl) and MIC/MBC Detection

*Pseudomonas* sp. was isolated from a formation water of the General Petroleum Company, Ras Gharib, Egypt on a Cetrimide Agar (Sigma-Aldrich, (St. Louis, MO, USA) at a salinity of 35,000 ppm (NaCl), purified, identified using 16S rRNA (data not shown), and deposited in GenBank (R301). The anti-bacterial activity of the synthesized HB(PDMA)s G_2_, G_3_, and G_4_ against the isolated SFB, *Pseudomonas* sp. (R301), at a salinity of 35,000 ppm was evaluated using the method of agar well (10 mm) diffusion as previously reported [23] on MHA at medium salinity (starting concentration of 10 mM). At the end of overnight cultivation at 37 °C, the clearing zone’s diameters were measured. All tests were performed three times, and the average values were recorded. The MIC values of the synthesized HB(PDMA)s G_2_, G_3_, and G_4_ were achieved by the micro-dilution method in 96-well microtiter plates as previously described [26,27]. The results were evaluated as above reported. MBC values of the synthesized HB(PDMA)s G_2_, G_3_, and G_4_ against isolated *Pseudomonas* sp. were estimated by taking 10 µL of the wells that showed no visible growth and were further sub-cultured onto the CA media at medium salinity. The agar plates were incubated at 37 °C for 20 h (i.e., until the growth was seen in the control plates). MBC values were defined as the corresponding concentrations of the synthesized HB(PDMA)s required for killing 99%.

### 2.7. Anti-Corrosion Activity of the Synthesized HB(PDMA)s

To evaluate the effect of the synthesized HB(PDMA)s G_2_, G_3_, and G_4_ as corrosion inhibitors for the cultivated *Pseudomonas* sp. (R301), a method was established using the batch experiments of the modified Nutrient Rich Simulated Seawater (NRSS) at medium salinity. The inocula for the corrosion experiment were enriched for 20 h at 37 °C using the CB media. The different reactors of HB(PDMA)s were established using mild steel coupons (AISI 1018 mild carbon steel strip measuring (1.5 cm × 1.5 cm × 0.32 cm) from COSASCO, Rohrback Cosasco Systems, Inc., Santa Fe Springs, CA, USA). The experiment was determined at MBC and 2XMBC for the isolated *Pseudomonas* sp. (R301) on the 12-well plates (Nunc GmbH & Co., Wiesbaden, Germany). Two control were performed in parallel to the corrosion and inhibition experiment: (i) the blank’s well (un-inoculated modified NRSS media) and (ii) the control’s well (inoculated with *Pseudomonas* sp. (R301) without the HB(PDMA)s). These mild steel coupons were first mechanically cleaned using emery papers of different grades, washed with distilled water and acetone, and then finally dried in a desiccator for further use. Furthermore, the weight loss was estimated (comparing the weight of the mild steel coupons before and after the experiment). From the weight loss results, the corrosion rate (g m^−2^ d^−1^) and the inhibition efficiency (%) of the metal corrosion were detected [33]. This experiment was then duplicated.

Scanning Electron Microscope (SEM, model Quanta 250 field emission gun, FEG, FEI company, Netherlands) at a magnification power of 25000×, and a gun with an acceleration voltage of 20 KV was used to confirm the corrosion inhibition efficiencies of the HB(PDMA)s at optimum concentrations against the isolated *Pseudomonas* sp. (R301) at a salinity of 35,000 ppm (NaCl). The mild steel coupons were obtained from the cultivated plate and directly washed with phosphate buffer saline (pH 7.4) for 5 min. Afterward, the coupons were fixed with a solution of 4% glutaraldehyde for 4 h and washed twice with PBS (5 min each), before washing with distilled water (5 min each). The coupons were then dehydrated using gradient concentrations of ethanol (25, 50, 75, and 100%) and kept dried in a desiccator.

## 3. Results

### 3.1. Synthesis and Characterization

The synthesized hyperbranched polymers showed good solubility in organic solvents like DMF and THF as well as in polar solvents like methanol and ethanol. The structure of the prepared hyperbranched polymers was confirmed by FT-IR, ^1^HNMR, and DLS.

**Figure 2:**
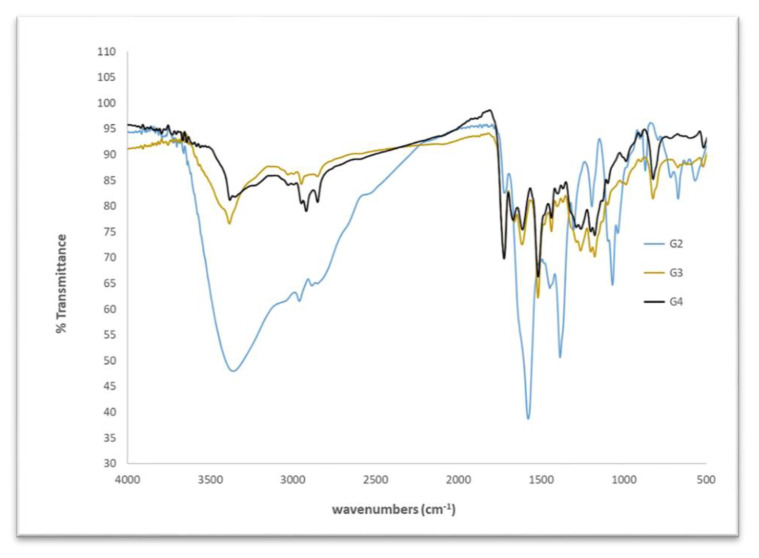
Fourier transform infrared (FTIR) spectrum of the synthesized HB(PDMA)s.

The FTIR of the prepared HB(PDMA)s G_2_, G_3_, and G_4_ (see Figure 2) detected the appearance of peaks at 1648 cm^−1^ and 1557 cm^−1^, which can be attributed to –CONH; a broad peak at 3200–3600 cm^−1^ assigned to the primary and secondary amine; a strong band at 3000–2849 cm^−1^ for the CH alkanes; and bands at 1124 cm^−1^ and 1173 cm^−1^assigned to the –C–N– stretches [34].

**Figure 3:**
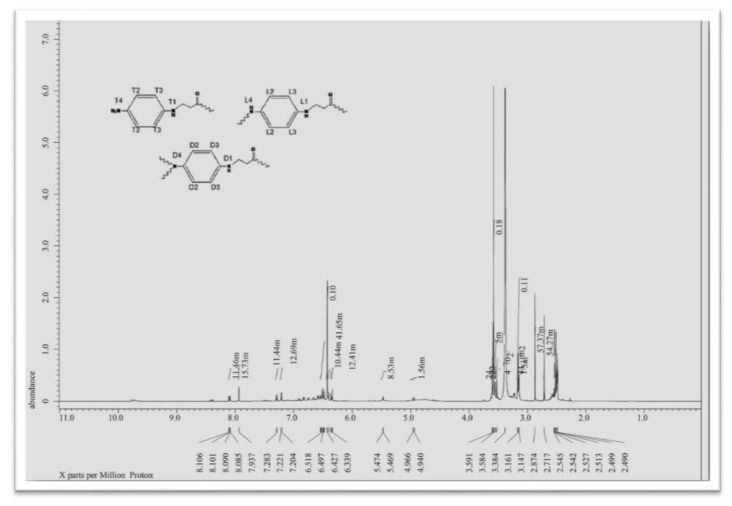
^1^HNMR spectrum of the synthesized HB(PDMA)G_3_.

The ^1^HNMR spectrum of HBPDMAG_3_ (see Figure 3, Figures S1 and S2), as a representative HB(PDMA) sample, showed signs of chemical shifts (δ, ppm, DMSO-d_6_) at 6.3–7.3 ppm corresponding to the aromatic protons; a singlet at 8.1 ppm corresponding to –NH proton; a singlet at 7.9 ppm attributed to –NH_2_; a singlet at 2.4–2.8 ppm corresponding to methylene groups; and a singlet at 3.5 ppm corresponding to –N–CH_2_ [35]. The prepared hyperbranched polymers G_2_, G_3_, and G_4_ had the same characteristic peaks.

**Figure 4:**
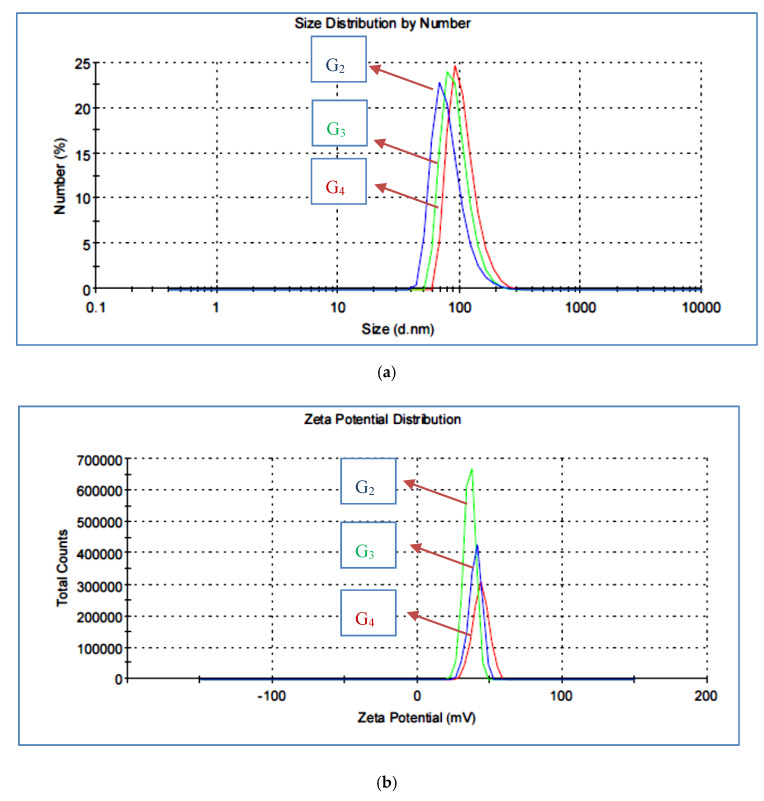
(**a**) Dynamic Light Scattering (DLS) images of the synthesized HB(PDMA)s G_2_, G_3_, and G_4_. (**b**) Zeta potential images of the synthesized HB(PDMA)s G_2_, G_3_ and G_4_.

The structure of a macromolecule can be widely studied by particle size and zeta potential [36]. The particle size distribution of the prepared HB(PDMA)s was measured by DLS and is illustrated in Figure 4a. The highly diluted solution of HB(PDMA) samples were dispersed in methanol, then in 2% diluted-distilled water with 0.01% HCl to protonate the terminal amine groups [29]. We could observe the particle sizes of HB(PDMA)s at.81.98, 93.86, and 107.1 nm of G_2_, G_3_, and G_4_, respectively. The branching number was increased by increasing the particle size. The zeta potential data are as shown in Figure 4b. was increased with increasing the number of branching leading to increasing the number of terminal amine groups as 35.8, 39.8, and 43.5 for HB(PDMA)G_2_, G_3_, and G_4_, respectively.

The molecular weights of the prepared HB(PDMA)s were determined by GPC. The concentrated solution of HBPDMA was dissolved in THF GPC grade, with a flow rate of 1 mL/min; the analysis achieved different molecular weights of Mn = 1820, 4300, and 9200 g mol^−1^.

The degree of branching (DB) “is one of the most characteristic parameters of hyperbranched polymers due to its correlation between the density of HBP structure and the number and position of the terminal groups.” DB is known as “the ratio of the molar fraction of branched and terminal groups relative to that of the total branching units” [37]. The AB_2_ polycondensation hyperbranched polymer is the most common type; buildings units may consist of initial (I), dendritic (D), terminal (T), and linear (L) units (see Figure 3). The calculation of the DB value can be estimated from the following equations [38,39]:

DB = (D+T)/(D+T+L)(1)

If the degree of polymerization (BP) is high, the number of T units is near that of D units. So, the above equation can be simplified as Equation (2):

DB = 2D/(2D+L) = 1/(1+L/2D)(2)

The DB of HBPs has been measured by direct and indirect approaches. The direct approaches include NMR spectroscopy measurements. Consider that the compound’s models are important and required to correctly assign the signals of L, D, and T units. The other one is the degradable method by chromatographic analysis, as described by Hawker [40] and Wooley [41], who used the degradable method to determine the DB of HBPs. Furthermore, the indirect viscometry method refers to the Mark–Houwink equation. $\left[\eta \right]=KM_{vis}^{\alpha }$ can be used to detect the DB, depending on the difference between the HBPs and the linear polymer in their intrinsic viscosity ([η]) and molecular weight (M_vis_) [42]. Obviously, the most common approach to detect the DB is by NMR spectroscopy. So, by using ^1^H NMR measurements, all the signals could be referred clearly to the T, D, and L units of the HB(PDMA)G_3_. The data recorded in Table 1 and illustrated in Figure 3 confirmed the structure and the calculated DB. The calculated values were in good agreement with HB(PDMA)G_3_ (DB = 66%).

### 3.2. Applications of the Synthesized HB(PDMA)s

In the present work, the HB(PDMA)s (Figure 1) were applied as broad-spectrum antimicrobial agents against standard microbial strains (two bacterial Gram-positive, one bacterial Gram-negative, and one yeast strains). The results (Table 2 and Figure S3) represented the broad antimicrobial activity of the synthesized HB(PDMA)s G_2_, G_3_, and G_4_ with the zone inhibitions ranging from 15.3–30.0 mm for the bacterial strains and 14.1–27.0 mm for the yeast strain in comparison to the positive control antimicrobial agents.

Overall, the HB(PDMA)s G_2_, G_3_, and G_4_ displayed antibacterial activity against both Gram-positive bacteria (20.0–30.0 mm) and Gram-negative bacteria (15.3–25.0 mm), as previously reported [43,44]. Moreover, the synthesized HB(PDMA)s G_2_, G_3_, and G_4_ showed inhibition activity against standard Gram-positive bacteria at lower MICs/MBCs ranging from 0.039–0.625/0.039–1.25 (mM) in comparison to Gram-negative bacteria (0.312–1.25/0.625–2.5 mM). Moreover, the synthesized HB(PDMA)s G_2_, G_3_, and G_4_ displayed MICs/MFCs ranging from 0.312–2.5/0.3212–2.5 mM. The highest antimicrobial activity effect of the HB(PDMA)s was attributed to HB(PDMA)G_4_ with inhibition zones of 28–30 mm, 25 mm, and 27 mm for the G+Ve, G−Ve, and candida strains, respectively, with the lowest MICs/MBCs and MICs/MFCs ranging from 0.039–0.078/0.039–0.156 mM, 0.312/0.625 mM, and 0.312/0.312 mM for the Gram-positive, for Gram-negative bacterial strains and yeast strain, respectively in comparison to HB(PDMA)G_2_ and HB(PDMA)G_3_ (Table 2 and Table 3). This result may be attributed to the number of branching as well as the increase in the functional groups and the bio-permeability action (size and molecular weight or lipophilic groups) [45].

The predicted mechanism of the broad antibacterial activity of HB(PDMA)s G_2_, G_3_, and G_4_ was related to the electrostatic interaction between the positively charged functional terminal-groups of HB(PDMA)s G_2_, G_3_, and G_4_ structure and the negatively charged bacterial surface phospholipid [46,47]. This interaction may slightly affect the cell’s selective permeability. Moreover, such interactions induce the protein’s denaturation of the membrane, leading to the destabilization of the cell membrane, leakage of the intracellular structure, and finally, the death of the bacterial cells [48]. Furthermore, the hypothesized antifungal activity of HB(PDMA)s G_2_, G_3_, and G_4_ was attributed to the electrostatic interaction between the cationic chain of the synthesized HB(PDMA)s and the negatively charged residues of the macromolecules of the fungal cell surface. This interaction induces leakage of intracellular electrolytes, which may alter the cell wall permeability and is essential to the enzyme system of fungal growth [11]. Overall, the effects of the functional group “*p-*phenylenediamine“ of HB(PDMA)s have been reputedly reported in many compounds as antimicrobial agents against bacteria and fungi [49,50].

Studies during the last century have provided substantial knowledge on microbial biofilms. Bacterial biofilms are considered as bacterial adhered or attached to biotic or abiotic surfaces, embedded in a complex (self-produced polymeric matrix), and they hard to eradicate with known biocides or antibiotics in comparison to their planktonic counterparts [51]. This may be due to their strong adherence to biotic or abiotic surfaces and/or their high resistance to antimicrobial agents. Therefore, one of the aims in this work was to study the anti-biofilm activity of HB(PDMA)s G_2_, G_3_, and G_4_ against the standard aerobic bacterial-developed biofilms of *B. subtilis* (ATCC 6633) and *E. coli* (ATCC 8739). The results reported in Table 4 display the minimum biofilm inhibitory concentrations (MBIC)s at a range of 5.0, 0.625, and 0.312 mM against *B. subtilis* developed biofilms and at a range of 5.0, 1.25, and 0.625 mM against *E. coli* developed biofilm for the synthesized HB(PDMA)s G_2_, G_3_ and G_4_, respectively. This result may be attributed to the number of branching, which reflects an increase in the reacted functional groups of the HB(PDMA)s, as previously reported [46,47]. This results in is agreement with the results reported by Labena and co-workers [27].

When bacterial biofilms develop on a metal, they present serious problems in the industry such as severe metal corrosion in comparison to their related planktonic counterparts. Therefore, one attempt of this work was to find a solution for the severity of microbial corrosion, especially those growing in a corrosive medium such as high salinity. *Pseudomonas* strains are Gram-negative, motile, rod-shaped bacteria, which are frequently observed in a marine-induced corrosion medium [52]. Many reports have focused on the importance of strains such as corrosive bacteria that have been cultivated and detected on carbon steels in a high salinity medium, which reflects severe damage and huge economic impacts [53,54,55]. Therefore, this work was to use the synthesized HB(PDMA)s G_2_, G_3_, and G_4_ as biocides and corrosion inhibitors for such corrosive bacteria at a high salinity medium of 35,000 ppm (NaCl). First, these synthesized compounds displayed antibacterial activity against the isolated and the enriched *Pseudomonas* sp. (R301) at a salinity of 35,000 ppm (NaCl) using the agar well diffusion method with the inhibition zones of 21, 40, and 44 mm for HB(PDMA)s G_2_, G_3_, and G_4_, respectively, in comparison to benzalkonium chloride (50 ppm) (see Table 5 and Figure S4).

The MIC/MBC results (Table 6) showed that the synthesized HB(PDMA)G_4_ had the lowest MIC/MBC values of 0.312/0.625 mM in comparison to HB(PDMA)s G_2_ and G_3_ with MIC/MBCs of 1.25/2.5, and 0.312/0.625 mM, respectively.

In order to examine the metal corrosion inhibition efficiency of these compounds on the carbon steels at the cultivated medium salinity, a batch experiment was developed. The results (Table 7 and Figure S5) displayed the mean metal corrosion rate for the blank wells (media with 35,000 ppm NaCl) and not-inoculated with *Pseudomonas* sp. R301) was 1.95 5 g m^−2^ d^−1^. These results can be attributed to the effect of the chemisorption of chloride ions on the metal. It has been reported that chloride anions can penetrate the oxide film that may have formed on the metal surface through pores or any defects. Then, they colloidally disperse or breakdown the formed oxide film and increase the permeability. Furthermore, when adsorbed on the metal surface, they favor hydration of the metal ions, increasing the corrosion probability and leading to pitting or crevice corrosion [56]. Moreover, such chloride anions can increase the metal corrosion rate by forming a self-perpetuating cycle, and the other free chloride acts as a corrosion catalyst. Equations (3)–(6) display the reactions between the mild steel and Cl_2_ ions [57]:

Fe → Fe^2+^ + 2e^−^ (3)

Fe^2+^ + 2H_2_O + 2Cl^−^ →Fe(OH)_2_ + 2HCl (4)

Fe(OH)_2_ + 3Cl^−^ → FeCl_3_ + 2OH^−^ + e^−^ (5)

FeCl + 3H_2_O → Fe(OH)_3_ + 3HCl (6)

However, the mean corrosion rate for the control wells (media with 35,000 ppm NaCl) and inoculated with *Pseudomonas* sp. R301) was 1.5 g m^−2^ d^−1^, with a corrosion inhibition efficiency of 22.8%. This result can be attributed to the effect of the isolated *Pseudomonas* sp. R301 biofilm, which covered the metal surfaces and protected them from the severity of the corrosion caused by salinity, as previously reported [58,59,60]. When the synthesized HB(PDMA)s G_2_, G_3_, G_4_ at MBCs and 2XMBCs were applied in the wells; the metal corrosion rates were reduced by increasing the concentrations of the HB(PDMA)s. The highest metal corrosion inhibition efficiency of 81.5% was achieved at a concentration of 5 mM for HB(PDMA)G_2_. However, HB(PDMA)G_3_ at a concentration of 1.25 mM reduced the corrosion rate and displayed a metal corrosion inhibition efficiency of 92.8%. Moreover, HB(PDMA)G_4_ exhibited the highest metal corrosion inhibition efficiency of 94.3% at a concentration of 0.625 mM in comparison to HB(PDMA)G_2_ and HB(PDMA)G_3_. The explanation for the effect of the HB(PDMA)s was attributed to the chemisorption and potentiality of the synthesized compounds to form passive films at the metal–polymer interface, and these reactions depend on the type of interaction between the metal and the synthesized molecules. Furthermore, the ligation capability of such synthesized molecules to a metal substrate plays an important role, which depends on the number of electronic charges and the chelating or active atoms of the synthesized compounds. Moreover, the highest metal corrosion inhibition efficiency of the synthesized HB(PDMA)G_4_ was attributed to more active centers and the highest possible sites for adsorption on the metal surface [61,62,63].

**Figure 5:**
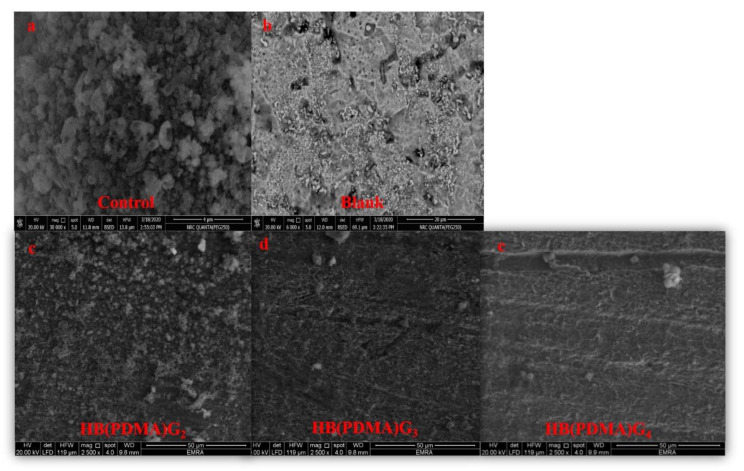
Scanning Electron Microscopy (SEM) images of the control (**a**); the metal surface with enriched *Pseudomonas* sp. (R301) at a salinity of 35,000 ppm (NaCl); the blank (**b**); the metal surface with a salinity of 35,000 ppm (NaCl); the synthesized HB(PDMA)s G_2_ (**c**); the synthesized HB(PDMA)s G_3_ (**d**); the synthesized HB(PDMA)s G_4_ (**e**) against the isolated and enriched *Pseudomonas* sp. (R301) at a salinity 35,000 ppm (NaCl) at the concentrations of 2XMBCs.

The corrosion inhibition efficiencies of the synthesized HB(PDMA)s against the isolated and enriched *Pseudomonas* sp. (R301) at the concentrations of 2XMBCs were confirmed using SEM (Figure 5).

## 4. Conclusions

HB(PDMA)s at different generations (G_2_, G_3_, G_4_) were successfully synthesized using AB_2_ monomers (*p-*phenylenediamine and methyl acrylate) via one-pot preparation as a commercial synthesis method in order to improve the reliability of the synthesis. Furthermore, the chemical structure of the synthesized HB(PDMA)s were successfully confirmed by FTIR and ^1^HNMR spectroscopy. The particle size of the synthesized HB(PDMA)s was increased by increasing the molecular weights and the number of branching from G_2_, G_3_, and G_4_. The HB(PDMA)s were applied successfully as broad-spectrum antimicrobial agents against Gram-positive, Gram-negative bacteria and *Candida albicans* strains. The synthesized HB(PDMA)G_4_ displayed the highest antimicrobial activity at low MICs/MBCs compared with HB(PDMA)G_3_ and HB(PDMA)G_2_. Furthermore, the HB(PDMA)s expressed anti-biofilm activity at low MBICs. In addition, HB(PDMA)s showed anti-bacterial effect against isolated and enriched *Pseudomonas* sp. (R301) that was cultivated at a salinity of 35,000 ppm (NaCl). The highest activity was by the synthesized HB(PDMA)G_4_ in comparison to HB(PDMA)G_3_ and HB(PDMA)G_2_ at the lowest MIC/MBC values. The HB(PDMA)s successfully displayed metal corrosion inhibition efficiencies of 52.5 and 81.5% for HB(PDMA)G_2_, 56.9 and 92.8% for HB(PDMA)G_3_, and 59.5 and 94.3% for HB(PDMA)G_4_ at the MBCs and 2XMBCs, respectively, in comparison to the negative control (un-inoculated media with a salinity of 35,000 ppm NaCl) and the positive control (inoculated with *Pseudomonas* sp. (R301), without the HB(PDMA)s).

## Figures and Tables

**Table 1:** The chemical shifts of Proton Nuclear Magnetic Resonance (^1^HNMR) for the synthesized HB(PDMA)G_3_ for the determination of D, L, and T units.

^1^H, ppm	Integration, m	Assignment	Functional Groups
8.106	11.46	D1	**–NH aromatic**
8.101	11.46	T1	
8.08	11.46	L1	
7.30	12.69	D2	**H aromatic**
7.27	11.44	D3	
6.42	41.56	T2	
6.36	10.42	L2	
6.34	8.43	L3	
6.33	12.44	T3	
3.56	40.24	T4	**–NH_2_ aromatic**
3.59	24.22	D4	**–N–CH_2_**
3.58	51.15 Wiesbaden	L4	

**Table 2:** The antibacterial activity of the synthesized HB(PDMA)s G_2_, G_3_, and G_4_. The results are described as the mean of the inhibition zone diameters (mm).

Compounds	*Staphylococcus aureus* (ATCC 29737)	*Bacillus subtilis* (ATCC 6633)	*Escherichia coli* (ATCC 8739)	*Candida albicans* (ATCC 10231)
	Mean Inhibition Zone (mm)*			
HB(PDMA)G_2_	20.0 ± 0.8	17.3 ± 0.2	15.3± 0.4	14.1 ± 0.2
HB(PDMA)G_3_	27.0 ± 0.4	28.0 ± 0.4	24.8 ± 0.6	20.6 ± 1.2
HB(PDMA)G_4_	28.0 ± 0.8	30.0 ± 0.0	25.0 ± 0.0	27.0 ± 0.0
**AMC	20.0 ± 0.0	17.0 ± 0.0	-	-
TE	-	-	22.0 ± 0.0	-
Flu	-	-	-	17.0 ± 0.2

*mm, *millimeters.* **AMC, Amoxicillin; TE, Tetracycline; Flu, Fluconazole (100 ppm).

**Table 3:** The Minimum Inhibitory Concentrations (MICs), Minimum Bactericidal Concentrations (MBCs), and Minimum Fungicidal Concentrations (MFCs) of HB(PDMA)s against different standard microbial strains.

Compounds	*Staphylococcus aureus* (ATCC 29737)		*Bacillus subtilis* (ATCC 6633)		*Escherichia coli* (ATCC 8739)		*Candida albicans* (ATCC 10231)	
	MIC *(mM)	MBC (mM)	MIC (mM)	MBC (mM)	MIC (mM)	MBC (mM)	MIC (mM)	MFC (mM)
HB(PDMA)G_2_	0.625	1.25	0.625	1.25	1.25	2.5	2.5	2.5
HB(PDMA)G_3_	0.078	0.156	0.156	0.625	0.625	1.25	0.625	0.625
HB(PDMA)G_4_	0.039	0.039	0.078	0.156	0.312	0.625	0.312	0.312

* mm, millimoles.

**Table 4:** The Minimum Biofilm Inhibitory Concentrations (MBICs) of HB(PDMA)s against different standard bacterial biofilms.

Compounds	*Bacillus subtilis* (ATCC6633)	*Escherichia coli* (ATCC 8739)
	MBIC (mM)	MBIC (mM)
HB(PDMA)G_2_	5.0	5.0
HB(PDMA)G_3_	0.625	1.25
HB(PDMA)G_4_	0.312	0.625

**Table 5:** The antibacterial activity of HB(PDMA)s against isolated *Pseudomonas* sp. (R301) at 35,000 ppm (NaCl).

Compounds	*Pseudomonas* sp. (R301)
	Mean Inhibition Zone (mm)
HB(PDMA)G_2_	20.6 ± 0.4
HB(PDMA)G_3_	40.0 ± 0.0
HB(PDMA)G_4_	44.0 ± 0.8
Benzalkonium chloride (50 ppm)	32.0 ± 0.0

**Table 6:** The MICs and MBCs of HB(PDMA) against isolated *Pseudomonas* sp. (R301) at 35,000 ppm NaCl.

Compounds	*Pseudomonas* sp. (R301)	
	MIC (mM)	MBC (mM)
HB(PDMA)G_2_	1.25	2.5
HB(PDMA)G_3_	0.312	0.625
HB(PDMA)G_4_	0.312	0.625

**Table 7:** The corrosion rate and inhibition efficiency (%) of HB(PDMA) at the MBCs and 2XMBCs of the isolated *Pseudomonas* sp. (R301) at 35,000 ppm (NaCl) in comparison to the blank.

Samples	Concentration (mM)	Mean Corrosion Rate (g m^−2^ d^−1^)	Inhibition Efficiency (%)
Blank	-	1.95 ± 0.03	0
Control	-	1.5 ± 0.10	22.8
HB(PDMA)G_2_	2.5	0.92 ± 0.05	52.8
HB(PDMA)G_2_	5.0	0.36 ± 0.01	81.5
HB(PDMA)G_3_	0.625	0.83 ± 0.05	57.4
HB(PDMA)G_3_	1.25	0.14 ± 0.005	92.8
HB(PDMA)G_4_	0.312	0.79 ± 0.02	59.5
HB(PDMA)G_4_	0.625	0.11 ± 0.02	94.3

## Supplementary Materials

The following are available online at https://www.mdpi.com/1996-1944/13/9/2076/s1, Figure S1: ^1^HNMR spectrum left shift of the synthesized HB(PDMA)G_3_, Figure S2: ^1^HNMR spectrum right shift of the synthesized HB(PDMA)G_3_, Figure S3: Photos show antimicrobial activity of the HB(PDMA)s at different generations (G_2_, G_3_, G_4_) against different standard microbial strains: (**a**) *Staphylococcus aureus* (ATCC 29737), (**b**) *Bacillus subtilis* (ATCC 6633), (**c**) *Escherichia coli* (ATCC 8739), and (**d**) *Candida albicans* (ATCC 10231) using a modified agar well diffusion method, Figure S4: Photos display antimicrobial activity of the synthesized HB(PDMA)s against the isolated and enriched *Pseudomonas* sp. (R301) at a salinity of 35,000 ppm (NaCl), Figure S5: Photo documenting the anti-corrosion activity of the synthesized HB(PDMA)s against the isolated and enriched *Pseudomonas* sp. (R301) at a salinity of 35,000 ppm (NaCl).
