# Supplementary material for: Progressive Applications of Hyperbranched Polymer Based on Diarylamine: Antimicrobial, Anti-Biofilm and Anti-Aerobic Corrosion
Source: Materials (Basel). 2020 Apr 30;13(9):2076. doi: 10.3390/ma13092076 (PMC7254357; doi:10.3390/ma13092076)
Supplement: Supplementary file 1 [file materials-13-02076-s001.pdf]

materials

MDPI

Supplementary Materials

Progressive Applications of Hyperbranched Polymer Based on Diarylamine: Antimicrobial, Anti-Biofilm and Anti-Aerobic Corrosion

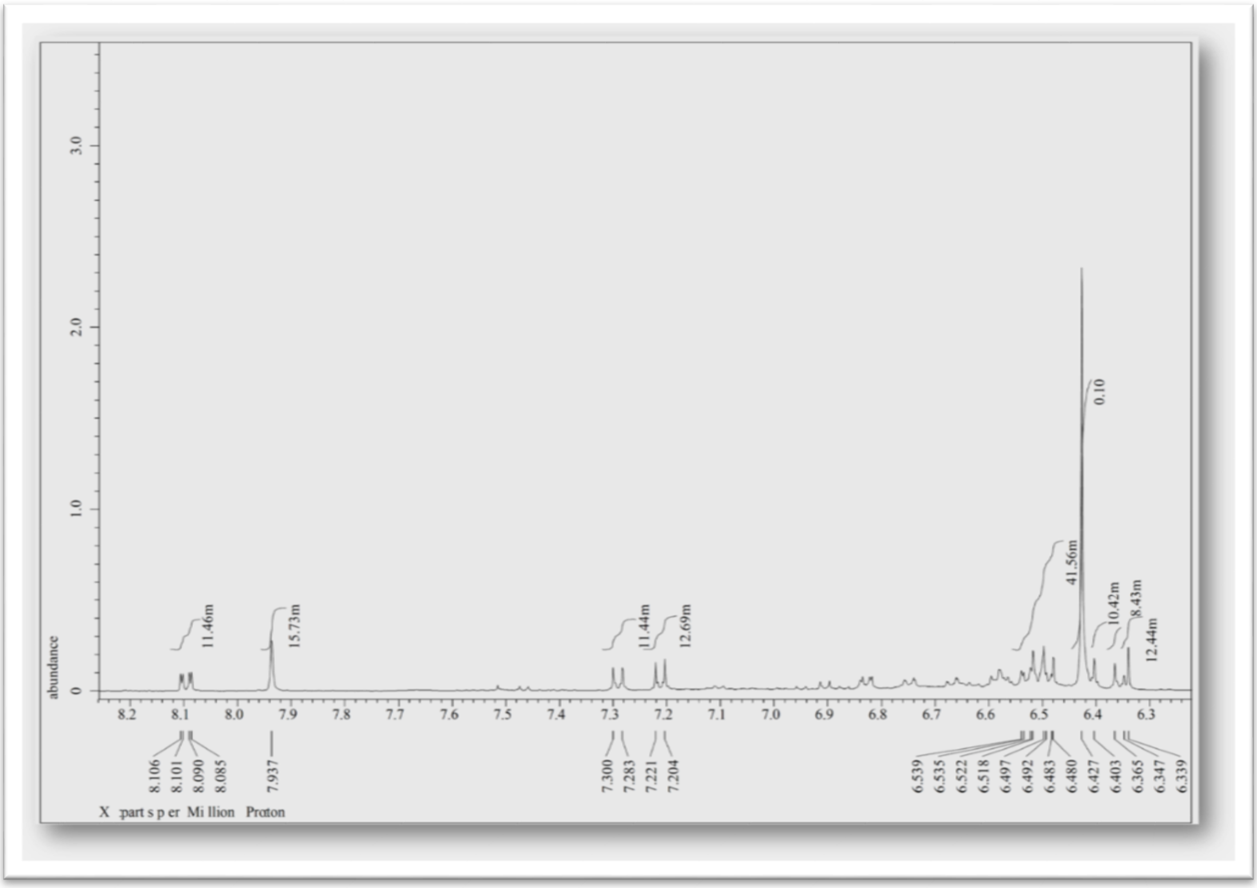

Figure S1. ^{1}HNMR spectrum left shift of the synthesized HB(PDMA)G_3.

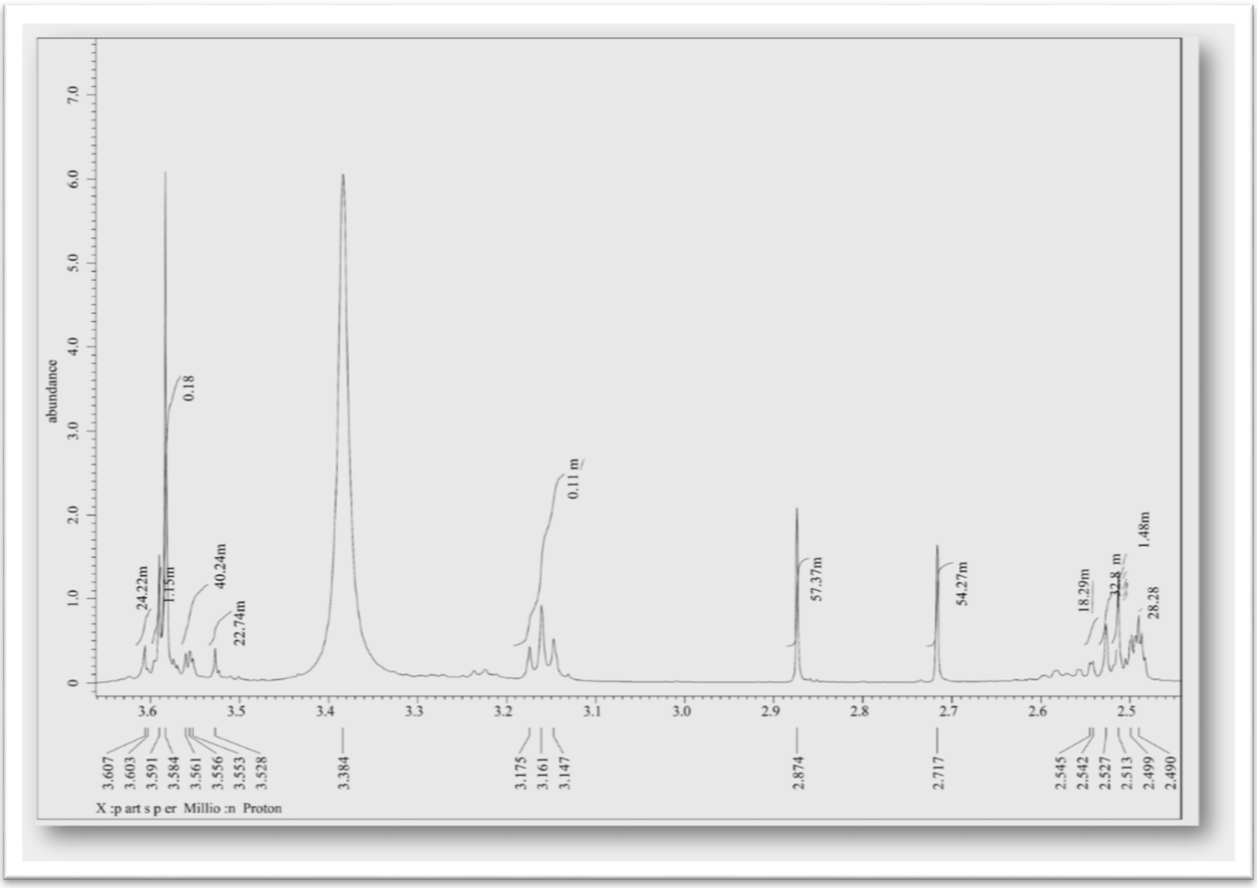

Figure S2. ^{1}HNMR spectrum right shift of the synthesized HB(PDMA)G_3.

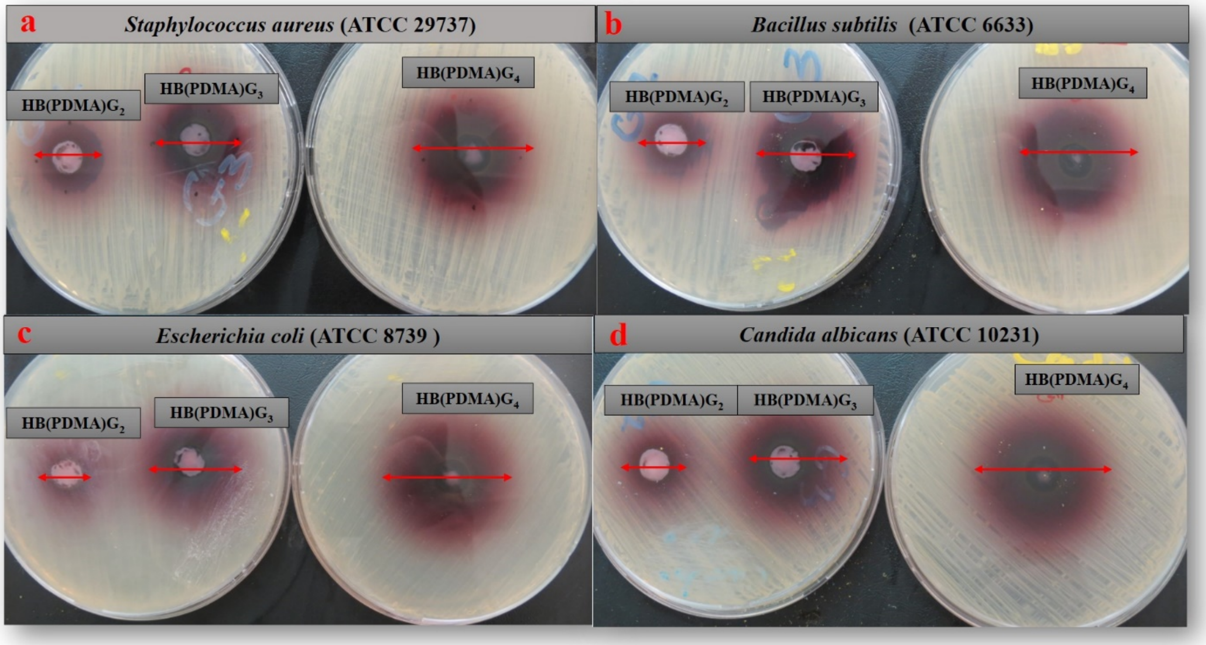

Figure S3. Photos show antimicrobial activity of the HB(PDMA)s at different generations (G2, G3, G4) against different standard microbial strains, (**a**) Staphylococcus aureus (ATCC 29737), (**b**) Bacillus subtilis (ATCC 6633), (**c**) Escherichia coli (ATCC 8739) and (**d**) Candida albicans (ATCC 10231) using a modified agar well diffusion method.

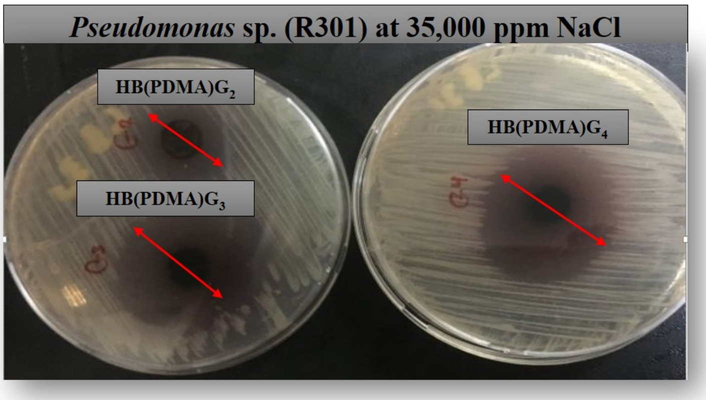

Figure S4. Photos display antimicrobial activity of the synthesized HB(PDMA)s against the isolated and enriched *Pseudomonas* sp. (R301) at a salinity of 35,000 ppm (NaCl).

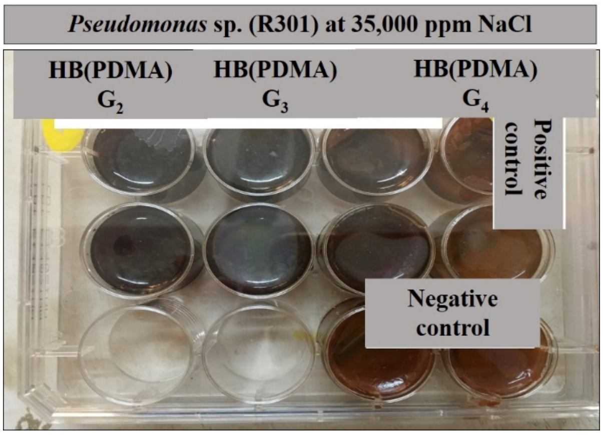

Figure S5. Photo documenting the anti-corrosion activity of the synthesized HB(PDMA)s against the isolated and enriched *Pseudomonas* sp. (R301) at a salinity of 35,000 ppm (NaCl).
